# Pilot evaluation of transcutaneous vagus nerve stimulation in chronic tinnitus: clinical outcomes and DTI insights

**DOI:** 10.1007/s00405-026-10066-6

**Published:** 2026-02-24

**Authors:** Özge Gedik Toker, Hilal Hüsam, Elif Kuru, Serdar Balsak, Nilüfer Bal, Remzi Doğan, Alpay Alkan, Orhan Özturan

**Affiliations:** 1 Department of Audiology, Faculty of Health Sciences, Bezmialem Vakif University, Istanbul, Turkey; 2 Department of Cognitive Neuroscience, Osnabrück University, Osnabrück, Germany; 3 Department of Radiology, Faculty of Medicine, Bezmialem Vakif University, Istanbul, Turkey; 4 Department of Otolaryngology, Faculty of Medicine, Marmara University, Istanbul, Turkey; 5 Department of Otolaryngology, Faculty of Medicine, Bezmialem Vakif University, Istanbul, Turkey

**Keywords:** Tinnitus, Neuromodulation, Vagus nerve, Diffusion tensor imaging

## Abstract

**Purpose:**

This study aims to evaluate the effectiveness of tVNS treatment in individuals with subjective chronic tinnitus.

**Methods:**

This pilot study included a total of 13 individuals (6 female and 7 male) aged 25–50 years (41.46 ± 8.04 years) who had complained of subjective tinnitus for at least two years. All participants underwent audiological evaluations, including assessments of tinnitus pitch, tinnitus loudness, and residual inhibition. Participants completed the Visual Analog Scale (VAS) for tinnitus severity, the Tinnitus Handicap Inventory (THI), the Beck Depression Inventory (BDI), and the Beck Anxiety Inventory (BAI). Additionally, DTI was performed, and both fractional anisotropy (FA) and apparent diffusion coefficient (ADC) values were calculated from the inferior colliculus, Heschl’s gyrus, lateral geniculate body, and lateral lemniscus regions. Participants received tVNS treatment with the Vagustim TENS device for 10 sessions (each 30 min long). Initial assessments were repeated after treatment.

**Results:**

After two weeks of tVNS treatment, significant improvement was observed in BDI and THI scores in individuals with subjective chronic tinnitus (*p* = 0.047 and *p* = 0.007, respectively). Furthermore, a significant decrease was observed in the FA values of the inferior colliculus (*p* = 0.001). However, no significant changes were detected in BAI scores, VAS, tinnitus pitch, tinnitus loudness, and residual inhibition (*p* > 0.05).

**Conclusion:**

The findings suggest that tVNS has potential benefit in reducing the daily burden of tinnitus and depression levels, and the effects of tinnitus on central auditory pathways may be modulated through neuroplastic changes. The results suggest that DTI can be utilized to assess the outcomes of tinnitus treatment. However, due to the small sample size and the absence of a control group, these results should be interpreted with caution. Further research is needed in this area.

## Introduction

Tinnitus refers to the perception of phantom auditory sensations in the absence of external acoustic stimuli. It is broadly categorized into two types: objective and subjective tinnitus. Objective tinnitus occurs when sound transmitted through body tissues reaches the ear and, unlike subjective tinnitus, can usually be heard by an observer using a stethoscope. Subjective tinnitus, on the other hand, is the perception of meaningless sounds unrelated to any physical sound and can only be heard by the person experiencing the tinnitus [1]. Subjective tinnitus is much more common than objective tinnitus. The prevalence of subjective tinnitus in the adult population is estimated to be between 10% and 15%, and prevalence increases with age [2]. Although many individuals report experiencing tinnitus, a substantial proportion do not find it distressing and therefore do not seek medical intervention. However, for others, tinnitus can severely impact quality of life, contributing to psychological conditions such as depression, anxiety, frustration, and insomnia [3]. Despite its significant socioeconomic burden, tinnitus remains a subjectively reported condition with no definitive diagnostic tests or standard treatment protocol [4]. Most tinnitus treatment options are aimed at alleviating or managing accompanying symptoms to make tinnitus less bothersome or less stressful [5].

One of the current tinnitus models defines the mechanism of tinnitus formation as “maladaptive plastic re-organization of the auditory cortex” and suggests that tinnitus may develop as a result of auditory deafferentation associated with peripheral hearing loss [6, 7]. Reduced input from the affected cochlear region may lead to decreased lateral inhibition from damaged frequency domains, which in turn may cause increased neural synchronization and hyperexcitability in the central auditory system [8]. Studies on Vagus Nerve Stimulation (VNS) and Transcutaneous Vagus Nerve Stimulation (tVNS), developed to reverse cortical plastic maladaptation associated with tinnitus, suggest that VNS may improve symptoms associated with tinnitus [9]. The vagus nerve is connected to the brainstem and limbic system, plays a significant role in sensory and autonomic functions, and provides a unique therapeutic input to the brain [10]. Functional imaging methods have demonstrated that VNS modulates activity in the auditory system, including the superior temporal gyrus, Heschl’s gyrus, planum polare, and planum temporale, as well as activity in the limbic system, particularly in the amygdala. Additionally, the parahippocampus is also modulated by VNS [9]. All these areas, which are thought to be modulated by VNS, are also associated with tinnitus [11].

Although the clinical efficacy of VNS is well known, the side effects associated with its invasiveness make this method difficult, risky, and expensive for widespread application in clinical populations [9]. To minimize these negative effects, transcutaneous stimulation of the afferent auricular branch (ABVN) of the vagus nerve in the outer ear has been evaluated as an alternative treatment option [12]. tVNS is a non-invasive, patient-friendly, low-cost neuromodulation method being researched for tinnitus treatment. The effect of tVNS on tinnitus treatment is based on its potential to modulate neuroplasticity in the auditory cortex and limbic system, thereby reducing the perception of tinnitus [13]. However, studies investigating the effectiveness of tVNS on tinnitus are quite limited in the literature. Furthermore, heterogeneity in electrode placement, stimulation parameters, and application protocols makes it difficult to compare results and generalize findings.

Diffusion Tensor Imaging is a neuroimaging method used to examine the connection patterns and microstructure of white matter tracts in the brain and to provide information about plastic/reactive changes in these connections. The most commonly used metric for quantifying the relationship between eigenvalues is fractional anisotropy (FA), a normalized scalar that represents the anisotropic portion of the diffusion tensor [14]. FA reveals information about fiber integrity and network reorganization, i.e., activity-dependent neuroplasticity [15]. The apparent diffusion coefficient (ADC) is another DTI parameter that measures the multidirectional isotropic diffusion of water. ADC is an expression of the diffusion amount and is calculated by taking the average of the three eigenvalues in the tensor [16]. DTI studies in individuals with tinnitus have increased in recent years to understand the neurobiology of tinnitus. Research into white matter differences associated with tinnitus has yielded a wide variety of inconsistent results to date. While white matter plasticity in auditory pathways has been reported in several studies [17–19], a few studies have also observed changes in limbic and auditory-limbic connections in the brain [18, 20].

This study aims to investigate the therapeutic efficacy of tVNS in individuals with chronic subjective tinnitus using objective and subjective assessment tools. As the first study to evaluate the effectiveness of tVNS on tinnitus using Diffusion Tensor Imaging (DTI), this study is expected to provide a unique contribution to the literature.

## Methodology

This study was conducted at the Department of Audiology, Bezmialem Vakif University. The Bezmialem Vakif University Non-Interventional Clinical Research Ethics Committee approved this study on 09.09.2021 (decision no: 2021/341). The study was supported by the Bezmialem Vakif University Scientific Research Projects Coordination Committee (date of contract: 22.11.2021, project number: 20211003). All participants were informed in detail about the study’s objectives, procedures, and possible outcomes. Informed consent was obtained from all individuals who voluntarily participated in the study, in accordance with the ethical principles outlined in the Declaration of Helsinki, before they participated in the research.

All participants included in the study underwent tympanometric and audiometric assessments, ten sessions of tVNS treatment, and tinnitus matching, residual inhibition, Diffusion Tensor Imaging (DTI), Visual Analog Scale (VAS), Tinnitus Handicap Inventory (THI), Beck Depression Inventory (BDI), and Beck Anxiety Inventory (BAI) assessments before and after tVNS treatment.

### Participants

The study included 18 participants (7 females and 11 males) aged 25–50 years with chronic subjective tinnitus who had no known neurological, metabolic, or systemic diseases. Five participants were excluded from the study because they were unable to continue with 10 sessions of tVNS treatment (due to logistical and/or time constraints). The study was completed with 13 participants (mean age: 41.46 ± 8.04 years; 6 females, 7 males). Participants had been experiencing tinnitus for a minimum of 2 years and a maximum of 18 years (mean duration: 8.33 ± 5.89 years). Five participants reported tinnitus in their right ear, two in their left ear, and six in both ears.

**Fig. 1:**
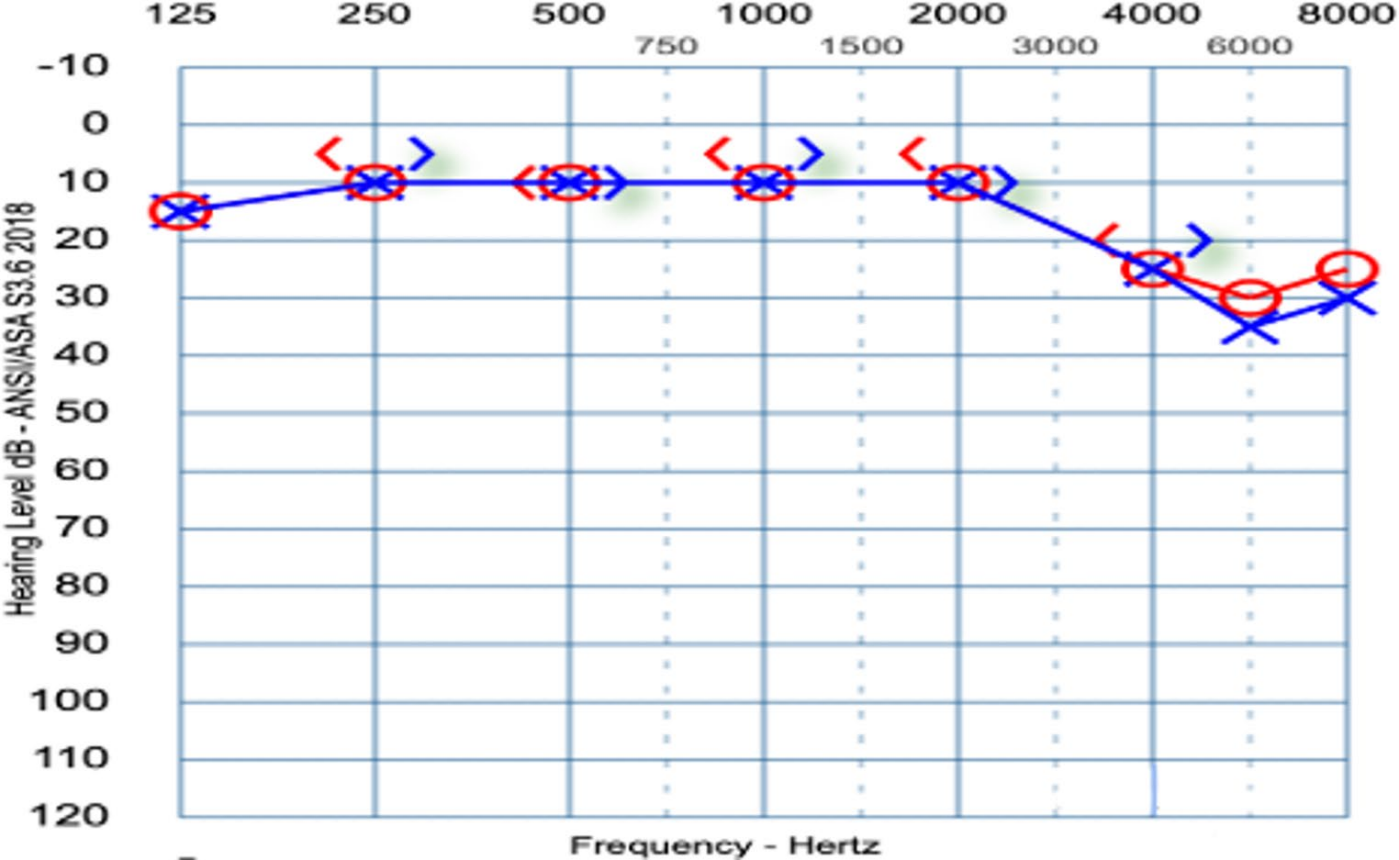
Mean audiometric thresholds of participants

All participants underwent tympanometric evaluation using the GSI Tympstar (Denmark) and audiometric evaluation using the Madsen Astera 2 audiometer (Otometrics^Ⓒ^, Denmark). Individuals with middle ear dysfunction observed in the tympanometric evaluation were excluded from the study. In the audiometric evaluation, air conduction thresholds were determined in a soundproof booth using Telephonics^®^ TDH-39 headphones (Telephonics, Farmingdale, NY, USA) between 125 and 8000 Hz, and bone conduction thresholds were determined using RadioEar B71 bone vibrator (RadioEar, New Eagle, Pennsylvania) between 250 and 4000 Hz. Figure 1 shows the average air and bone conduction thresholds for the 13 participants included in the study.

### Transcutaneous vagus nerve stimulation (tVNS)

The Vagustim Transcutaneous Electrical Nerve Stimulation (TENS) device (Vagustim Health Technologies Inc, Turkey) was used for tVNS treatment. The Vagustim TENS device is a CE-certified device routinely used in pain treatment that provides stimulation through electrodes applied to the skin. This study is the first to investigate the effectiveness of the Vagustim TENS device, which has a similar mechanism of action to other transcutaneous vagal stimulation devices (CM02, STV02, NEMOS, VITOS, and TENS-200, etc.), when used in the treatment of tinnitus.

**Fig. 2:**
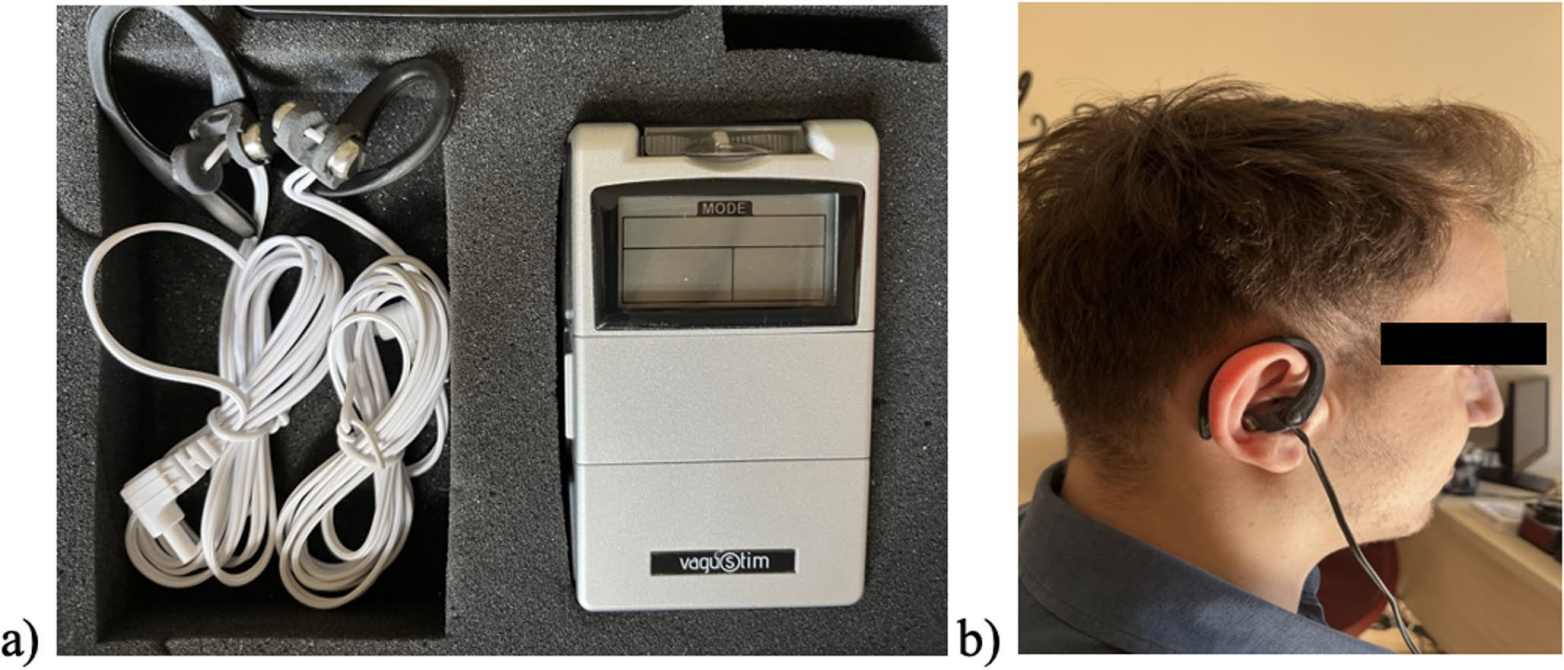
The tVNS Stimulation Setup and Electrode Placement. Panel (**a**) shows the device used to deliver transcutaneous vagus nerve stimulation. Panel (**b**) illustrates the standardized placement of electrodes on the left tragus. These images describe the stimulation protocol applied during the study. tVNS: Transcutaneous Vagus Nerve Stimulation

Electrical stimulation was bilaterally applied to the inner surface of the tragus and concha regions using electrodes specially manufactured according to the size of the ear **(**Fig. 2**)**. Studies on ear anatomy have shown that the tragus, concha, and cymba concha are locations in the human body where cutaneous afferent vagus nerve distributions are found. tVNS approaches involve various devices and stimulation protocols, and there is still a lack of robust data on the location and type of stimulation required to achieve an effect. In the present study, the frequency of the current applied by the device was set at 10 Hz and the pulse duration was fixed at 300 µs. The module was applied in TENS mode and with a biphasic asymmetric waveform. The amplitude was slowly increased for each ear separately to the level at which the patient felt the current and was not uncomfortable. Participants received 30 min of t-VNS treatment for 10 sessions over two weeks.

### Tinnitus pitch and loudness matching

Tinnitus mapping was performed on all participants using an Otometrics Madsen Astera 2 audiometer in a soundproof booth to determine tinnitus pitch and loudness. For tinnitus pitch matching, a 1 kHz pure tone target was first presented. The patient was asked to evaluate the similarity of the target sound to their tinnitus in terms of pitch. Based on the participant’s feedback, the target sound was changed in 1/2 octave steps and then in 1/6 octave steps. The same procedure was repeated until the participant was satisfied with the pitch similarity between the target and tinnitus. To match the tinnitus sound intensity, the target sound was presented at a low intensity level (close to the threshold level) at the tinnitus frequency, and the sound intensity was changed in 5 dB steps, followed by 1 dB steps, until the patient found the sound intensity similar to the tinnitus.

### Residual Inhibition (RI) assessment

After tinnitus pitch and loudness matching, residual inhibition was evaluated. First, the Minimum Masking Level (MML), the intensity at which the tinnitus sound is inaudible at the same frequency as the patient’s tinnitus, was determined. The MML intensity was increased by 10 dB and presented to the participant’s tinnitus ear. In individuals with bilateral tinnitus, the stimulus was sent to the ear where tinnitus was perceived most intensely. The stimulus was delivered for 60 s via TDH 39 headphones using an Otometrics Madsen Astera 2 audiometer. After the stimulus was stopped, the participant was asked to listen to their tinnitus and respond whether the tinnitus had completely disappeared (complete inhibition), decreased (partial inhibition), or remained unchanged (no inhibition).

### Patient-Reported outcome Measures – PROMs

Patient-reported outcome measures (PROMs) were used to assess the severity of symptoms associated with tinnitus and psychological status. For this purpose, the Visual Analog Scale (VAS), Tinnitus Handicap Inventory (THI), Beck Depression Inventory (BDI), and Beck Anxiety Inventory (BAI) were assessed before starting tVNS treatment and after 10 sessions of treatment.

To assess tinnitus severity, patients were asked to rate their subjective perception of tinnitus severity using the VAS. The VAS is a measure frequently used by various disciplines for all types of pain and has several versions. It has been adapted and used for tinnitus in many studies [21]. In the VAS questions, participants marked their subjective tinnitus severity level on a scale numbered from 0 to 10 (“0” represents the lowest and “10” the highest level).

The THI is a self-report questionnaire commonly used in studies to assess the impact of tinnitus on daily life. The Beta version of the questionnaire, which has been validated for Turkish by Aksoy et al. (2007), was used in the study [22]. The THI consists of 25 items and 3 subscales (emotional, functional, and catastrophic). Survey questions are answered as “Yes” (4 points), “Sometimes” (2 points), or “No” (0 points). A minimum of 0 points and a maximum of 100 points can be obtained from the questionnaire. 0–16 points is considered slight or no handicap, 18–36 points is mild handicap, 38–56 points is moderate handicap, 58–76 points is severe handicap, and 78–100 points is catastrophic handicap [22, 23].

The BAI is a 21-item, self-reported tool used to assess the severity of anxiety over the past seven days. It measures the physical, emotional, and cognitive aspects of anxiety, as well as the fear of losing control [24]. A Turkish validity and reliability study was conducted by Ulusoy et al. (1998) [25]. Each item is rated on a 4-point scale ranging from 0 (not at all) to 3 (severely—I could barely stand it). Based on the total score, anxiety is classified as minimal (0–7), mild (8–15), moderate (16–25), and severe (26–63) [24, 25].

The BDI is a 21-item self-report assessment tool used to evaluate depressive symptoms over the previous two weeks [26]. Kapci et al. (2008) conducted a Turkish validity and reliability study [27]. Each item is rated on a 4-point scale ranging from 0 (“absent”) to 3 (“severe”). Based on the total score, depression is classified as minimal (0–13), mild (14–19), moderate (20–28), or severe (29–63) [26, 27].

### Diffusion tensor imaging (DTI)

Magnetic resonance imaging (MRI) was performed using a 1.5 Tesla scanner (Avanto, Siemens Healthineers, Erlangen, Germany). Cranial MRI and DTI were obtained without the use of any invasive diagnostic procedures. Prior to DTI analysis, the identified white matter tracts were visually examined by an experienced radiologist.

Imaging protocol included axial T1-weighted sequences (TR/TE: 500/80 ms), axial T2-weighted sequences (TR/TE: 3770/90 ms), sagittal T2-weighted sequences (TR/TE: 5700/90 ms), and post-contrast T1-weighted sequences (TR/TE: 350/8 ms). DTI data sets were acquired using spin-echo echo-planar imaging (SE-EPI) sequences with the following parameters: 5 mm slice thickness, 230 mm field of view (FOV), 30 diffusion gradient directions, 6000 ms repetition time (TR), 85 ms echo time (TE), and two b values (b = 0 s/mm² and b = 1000 s/mm²) were used. The image matrix size is 128 × 128.

**Fig. 3:**
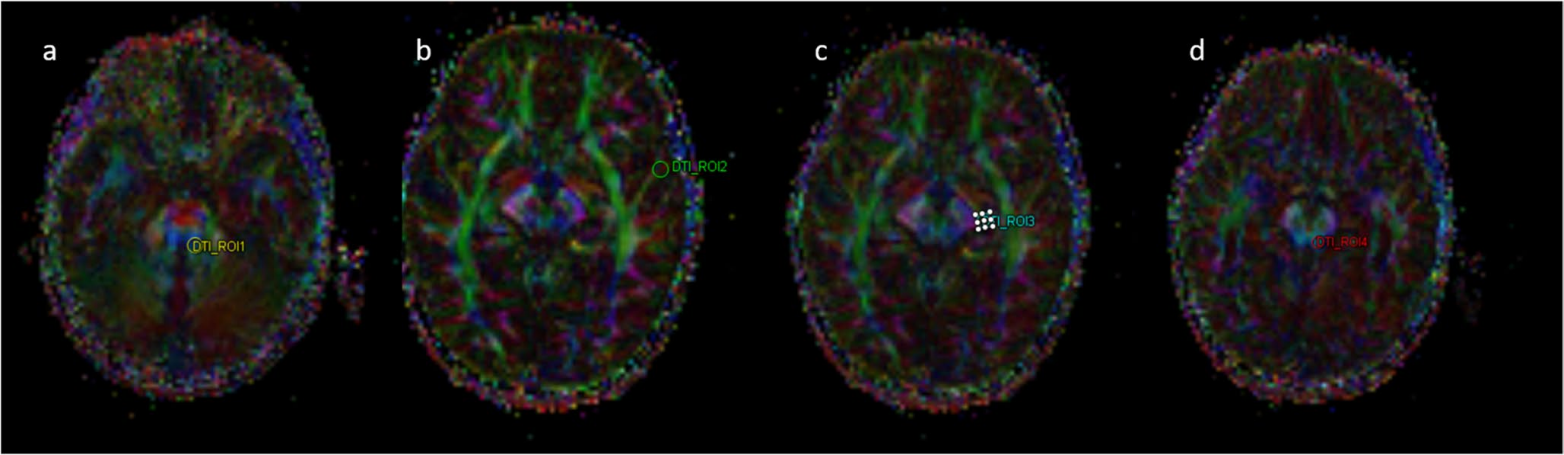
An exemplifier case for region of interest (ROI) samplings from color-coded FA map images. Placement of the ROIs on the inferior colliculus (**a**), Heschl’s gyrus (**b**), lateral geniculate body (**c**), and lateral lemniscus (**d**) on a directionally color-coded map and anisotropy map. These ROIs were used to extract pre- and post-tVNS diffusion metrics for exploratory comparison

A region-of-interest (ROI)-based method was used for DTI assessment. Diffusion metrics, including fractional anisotropy (FA) and apparent diffusion coefficient (ADC), were calculated using the Siemens Syngo-Via workstation (version 2.0). ROIs were manually drawn as circular areas (approximately 10–20 mm²) on color-coded FA maps, placed over anatomically predefined regions. These regions included the inferior colliculus (IC), Heschl’s gyrus (HG), lateral geniculate body (LGB), and lateral lemniscus (LL) pathway. A representative FA map showing the ROI placement is shown in Fig. 3.

### Statistical Analysis

Statistical analyses were performed using IBM SPSS Statistics version 24.0. Descriptive statistics, including mean, standard deviation, minimum, and maximum values, were calculated for the study variables. The normality of the distribution of continuous numerical data was assessed using the Shapiro–Wilk test. To compare pre- and post-treatment results, the paired-samples t-test was applied to variables showing a normal distribution, while the Wilcoxon signed-rank test was used for variables not showing a normal distribution. No correlation analyses were conducted between clinical outcomes and FA/ADC changes due to the small sample size (*n* = 13), which limits the statistical power and increases the risk of spurious associations. All results were interpreted within a 95% confidence interval, and statistical significance was set at *p* < 0.05.

## Results

Following 10 sessions of tVNS treatment, BDI and THI scores significantly decreased (*p* = 0.047, *p* = 0.007, respectively). However, no significant difference was observed in BAI scores (*p* = 0.273). The results are shown in Table 1.

**Table 1:** Changes in BDI, BAI, and THI scores following tVNS treatment

	Pre-tVNS (*n* = 13)				Post-tVNS (*n* = 13)				*p*
	Mean	SD	Min	Max	Mean	SD	Min	Max	
BDI	14.15	5.30	7	25	12.15	6.31	2	22	0.047*
BAI	8.85	5.11	0	17	7.85	5.44	0	19	0.273
THI	49.54	18.24	26	86	40.92	23.77	10	92	0.007*

*p < 0.05, *BDI *Beck depression inventory, *BAI *Beck anxiety inventory, *THI *Tinnitus handicap inventory,* tVNS *transcutaneous vagus nerve stimulation

**Fig. 4:**
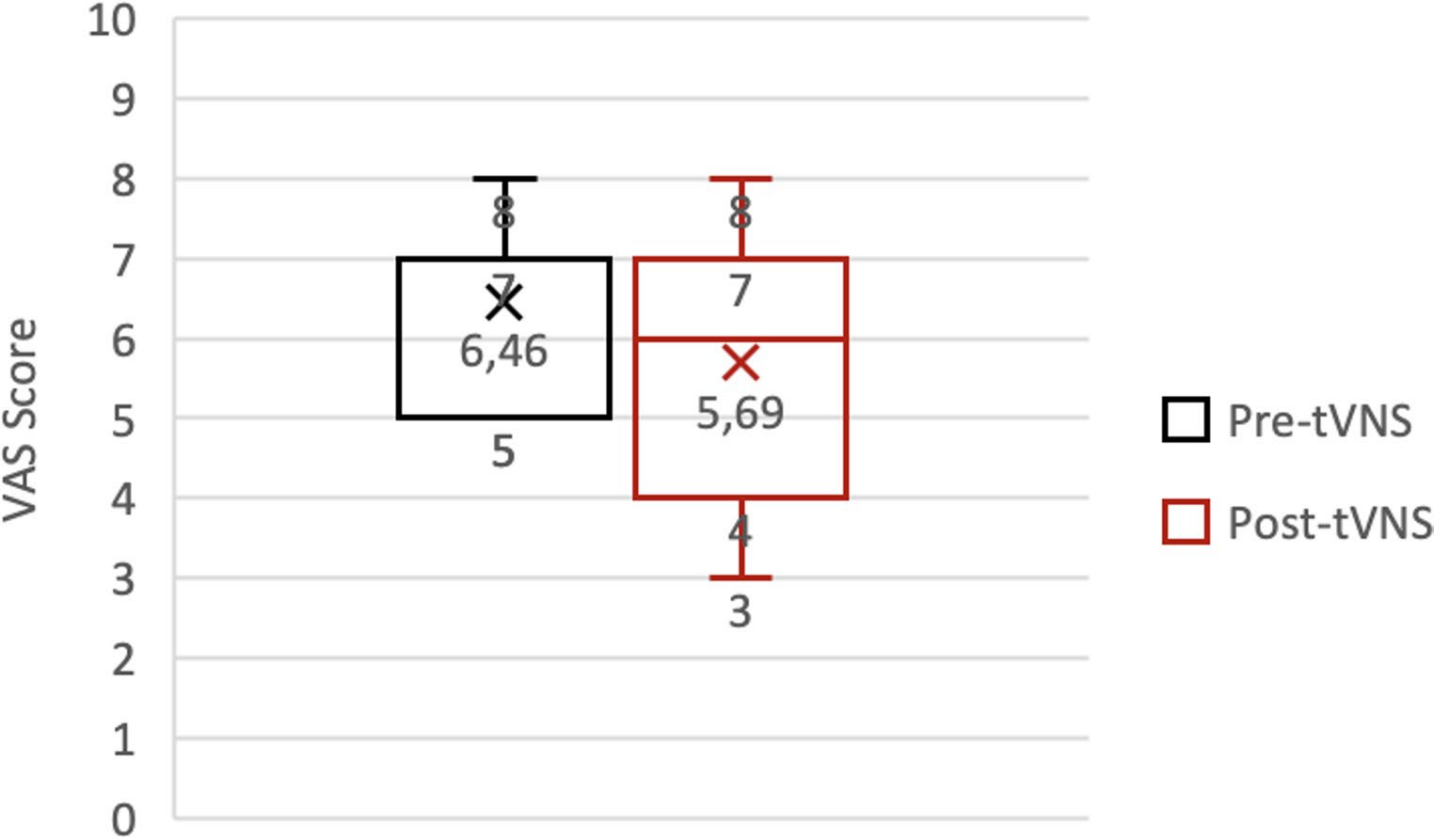
VAS scores pre- and post-tvns treatment. tVNS: Transcutaneous Vagus Nerve Stimulation

There was no statistically significant difference in Tinnitus Pitch, Tinnitus Loudness, and Residual Inhibition before and after tVNS treatment (*p* > 0.05). Tinnitus pitch and loudness matching could not be performed in 2 participants, and thus, residual inhibition could not be assessed. The results are shown in Table 2. Tinnitus severity, assessed using the VAS score, decreased in 7 participants after treatment, remained the same in 5 participants, and increased in 1 participant. Although a decrease in the mean VAS score was observed after treatment, this difference did not reach statistical significance (*p* = 0.119, Fig. 4).

**Table 2:** Comparison of Tinnitus-Related measures Pre- and Post-tVNS treatment

		Patient													*p*
		1	2	3	4	5	6	7	8	9	10	11	12	13	
Pitch (kHz)	Pre	8	9	7.1	8	6.15	0.125	11.2	5	10	4	0.5	∅	∅	0.879
	Post	7.1	5.6	8	8	7.5	0.14	11.2	8	7.1	4.5	1	∅	∅	
Loudness (dB HL)	Pre	70	50	55	75	52	50	35	70	25	55	20	∅	∅	0.652
	Post	65	70	66	55	38	50	45	60	20	55	15	∅	∅	
Residual Inhibition (-/+)	Pre	-	-	-	-	-	+	-	+	-	-	-	∅	∅	0.250
	Post	+	+	-	-	+	+	-	+	-	-	-	∅	∅	
VAS Score	Pre	8	5	5	5	8	7	5	6	7	7	7	7	7	0.119
	Post	5	4	3	5	8	4	8	4	6	7	6	7	7	

*tVNS *transcutaneous vagus nerve stimulation, *VAS *visual analog scale

A significant decrease was observed in the Inferior Colliculus FA values assessed with DTI (*p* = 0.001). The value, which was 0.733 ± 0.094 before treatment, decreased to 0.608 ± 0.055 after treatment. No significant changes were observed in other DTI measurements (Table 3).

**Table 3:** DTI metrics Pre- and Post-tVNS treatment

DTI	Pre-tVNS (*n* = 13)				Post-tVNS (*n* = 13)				*p*
	Mean	SD	Min	Max	Mean	SD	Min	Max	
LGB_ADC	1.140	0.151	0.932	1.465	1.189	0.195	0.915	1.733	0.311
LGB_FA	0.383	0.058	0.301	0.472	0.347	0.077	0.212	0.488	0.256
HG_ADC	0.922	0.069	0.758	1.027	0.898	0.041	0.853	1.005	0.162
HG_FA	0.245	0.048	0.168	0.312	0.246	0.055	0.142	0.320	0.940
IC_ADC	0.863	0.120	0.615	1.097	0.897	0.135	0.716	1.194	0.310
IC_FA	0.733	0.094	0.567	0.863	0.608	0.055	0.489	0.704	0.001*
LL_ADC	0.927	0.133	0.677	1.198	1.037	0.198	0.765	1.403	0.221
LL_FA	0.573	0.052	0.487	0.676	0.552	0.056	0.430	0.614	0.271

*p < 0.05, *DTI *diffusion tensor imaging, *tVNS *transcutaneous vagus nerve stimulation, *ADC *apparent diffusion coefficient (x10^− 3^mm^2^/s), *FA*: fractional anisotropy, *LGB *lateral geniculate body, *HG *Herschel’s gyrus, *IC *inferior colliculus, *LL *lateral lemniscus

**Fig. 5:**
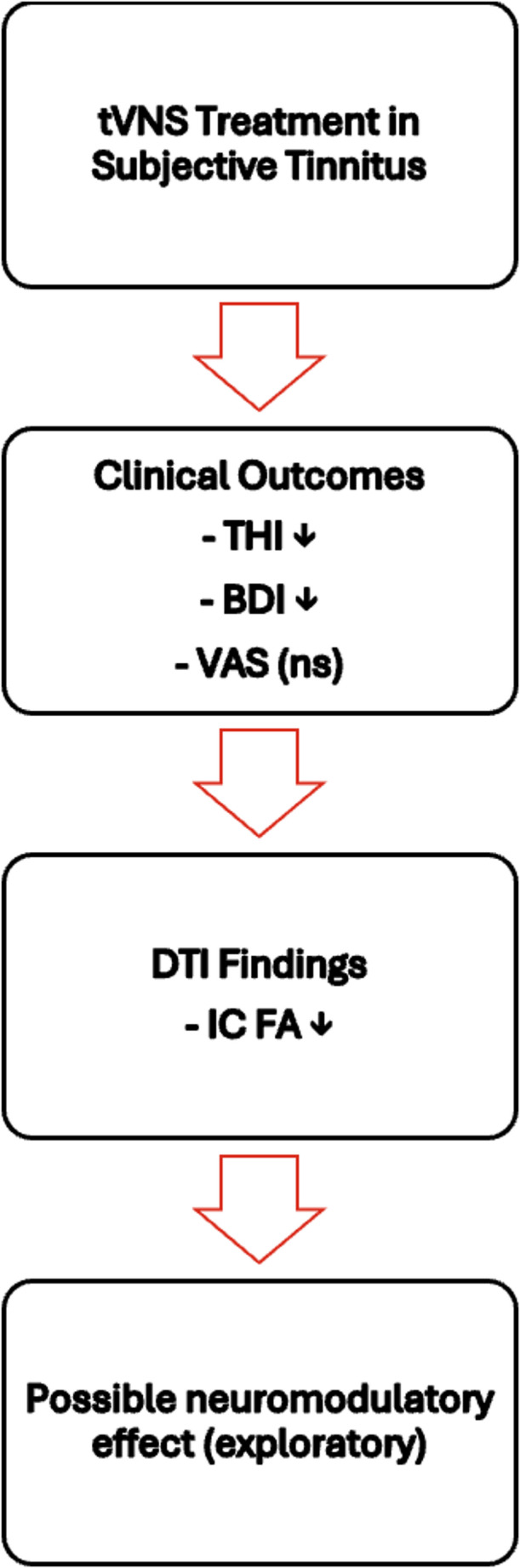
Summary diagram showing clinical outcomes and neuroimaging findings following tVNS treatment. tVNS: Transcutaneous Vagus Nerve Stimulation, THI: Tinnitus Handicap Inventory, BDI: Beck Depression Inventory, VAS: Visual Analog Scale, ns: non-significant, DTI: Diffusion Tensor Imaging, IC: Inferior Colliculus, FA: fractional anisotropy

To provide an integrated overview of clinical and neuroimaging changes, a summary figure has been created **(**Fig. 5**).** Consistent with statistical analyses, decreases in THI and BDI scores were presented alongside decreases in inferior colliculus FA values. As a control or sham-tVNS group was not included, these relationships cannot be interpreted causally; rather, they highlight potential neuromodulatory models that require further investigation in future controlled studies.

## Discussion

This study evaluated the potential benefit of tVNS treatment in participants with chronic subjective tinnitus. Following ten sessions of tVNS treatment, a decrease in participants’ depression levels and an improvement in the negative effects of tinnitus on daily life were observed. These findings are supported by decreased scores on the BDI and THI. Furthermore, DTI findings suggest that tVNS may be associated with changes in white matter integrity in the inferior colliculus.

A non-invasive tVNS method applied to the auricular branch of the vagus nerve (ABVN) has recently been introduced [28]. Functional magnetic resonance imaging (fMRI) studies have revealed deactivation of the limbic system (amygdala, hippocampus, and parahippocampal gyrus) following tVNS [29]. While tVNS is used in various pathologies, including epilepsy [30], depression [31], pain [32], and migraine [33], studies related to tinnitus began with the promising pilot study by Lehtimaki et al. (2013). In the study, magnetoencephalography (MEG) was performed while applying tVNS to eight tinnitus patients; additionally, classical music with notes specifically tailored to the tinnitus frequency was continuously presented under both tVNS-on and tVNS-off conditions. The amplitude of N1m responses decreased following tVNS application [34]. In another MEG study, tVNS efficacy was evaluated using the same stimulation protocol, and the authors concluded that tVNS successfully modulated beta and gamma band activity associated with tinnitus and therefore holds potential for tinnitus treatment [35].

Kreuzer et al. (2014) evaluated the feasibility, safety, and efficacy of tVNS treatment in individuals with chronic tinnitus and demonstrated the long-term feasibility of tVNS in individuals with chronic tinnitus. As a result of the study, Tinnitus Questionnaire (TQ) and BDI scores decreased significantly following tVNS treatment [36]. Similarly, Suk et al. (2018) reported that VAS scores, THI, and BDI scores related to tinnitus improved significantly four weeks after the completion of tVNS treatment [37]. Our study has shown that a two-week tVNS treatment may be sufficient for significant improvement in BDI and THI scores. Patients with tinnitus-related mental stress (TRMS) may also benefit from tVNS. In TRMS patients, increased sympathetic activity is accompanied by ANS imbalance and, consequently, reduced parasympathetic function. A study has shown that acute tVNS application increases parasympathetic activity. The authors suggested that tVNS should be used not as a standalone treatment but as a complement to a treatment program that aims to correct sympathetic-vagal imbalance through parasympathetic activation of all its components [38].

Studies have reported significantly higher rates of accompanying anxiety, depression, and low self-esteem in tinnitus patients. However, it has been suggested that the tendency toward tinnitus may cause inappropriate activation of the limbic and sympathetic components of the autonomic nervous system [39]. In the current study, a significant decrease was observed in THI scores, which measure the effects of tinnitus on quality of life, and in BDI scores, which assess depression levels, following treatment. However, no significant change was detected in BAI scores, which assess anxiety levels. This finding suggests that tVNS may have a potential benefit, particularly in regulating mood and reducing the burden of tinnitus on daily life but may have had a limited effect on anxiety symptoms. The literature also reports that tVNS has shown positive effects on depressive symptoms, but its effects on anxiety are more uncertain [40]. One reason for this situation may be that the BAI focuses primarily on somatic anxiety symptoms (e.g., palpitations, sweating, dizziness) [41]. Therefore, while tVNS’s effect on mood regulation via the limbic system may lead to measurable improvement in depression levels, it may have a more limited effect on physical anxiety symptoms. In future studies, the use of scales that include cognitive and emotional dimensions in addition to the BAI for assessing anxiety will contribute to a more comprehensive understanding of the effects of tVNS.

DTI studies have revealed differences in FA and structural connectivity in cases of tinnitus, hearing loss, and both conditions occurring together, demonstrating the existence of neural networks specific to tinnitus [42]. Along with this, in some studies, increased FA values were observed in the white matter of the left thalamic, frontal, and parietal regions in individuals with tinnitus [17, 43], while others have observed a decrease in FA values, suggesting that this may be a reflection of underlying microstructural changes and/or dysfunction of associated fibers in tinnitus patients [20, 44]. FA increase, on the other hand, can be attributed to various factors such as increased myelination, decreased axonal diameter, increased packing density, and decreased branching [43].

The ADC value primarily reflects changes in myelination, neural plasticity, and alterations in nerve fiber density [17]. Gunbey et al. (2017) reported a significant increase in ADC values in regions such as the inferior colliculus (IC), medial geniculate body (MGB), thalamic reticular nucleus (TRN), and amygdala (AMG) in tinnitus patients [20]. Another study also observed significantly higher mean diffusivity (MD) values in the auditory cortex, amygdala, and arcuate fasciculi regions in the tinnitus group compared to the control group [45]. It has been suggested that these observed changes may be the result of differences in axonal packing density caused by demyelination, axonal loss, or tinnitus [46]. Seydell-Greenwald et al. (2014) also reported FA and mean diffusion (MD) changes in the auditory cortical white matter associated with tinnitus in relation to hearing loss and age [17]. There are differences between studies in terms of hearing loss, age, and the regions evaluated, and the results are unclear as to whether these changes are caused by tinnitus or hearing loss [46]. No noteworthy post-treatment ADC value changes were observed in our study. Several factors can explain this finding. The small sample size (*n* = 13) may limit statistical power, and the limited treatment duration (two weeks) may be insufficient for microstructural diffusion changes to emerge. Furthermore, the effects of tVNS may be more sensitive to parameters measuring anisotropy, such as FA, and may not produce significant changes in mean diffusion (ADC/MD). Our study is the first to evaluate the efficacy of tVNS using DTI, so further studies are required in this area.

The inferior colliculus is the primary relay center for auditory signals originating from the cochlear nucleus. It is located in the midbrain and plays a crucial role in the integration of auditory signals and in frequency and pitch processing. Melcher et al. (2000) detected abnormal activity in the IC of patients with tinnitus using fMRI [47]. To evaluate the potential effects of IC stimulation on tinnitus, Offutt et al. (2014) compared the degrees of suppression and facilitation that occur in the IC with different stimulation paradigms. Using a guinea pig model, they found that stimulation of the dorsal IC caused both suppressive and facilitative changes throughout the central IC, which could occur during stimulation and persist after stimulation. Interestingly, stimulation of the dorsal IC induced greater suppression when paired with broadband noise stimulation. Although the study did not use animals with induced tinnitus, it demonstrated that stimulation of the dorsal IC can induce plasticity and accompanying activity changes within the central IC, which may be applicable in tinnitus treatment [48].

In the current study, a significant decrease was observed only in IC-FA values among the regions evaluated with DTI. It is previously known that increased bursting activity, neural synchronization, and tonotopic reorganization in IC have been observed in individuals with tinnitus [49]. In this context, it is thought that tVNS may suppress excessive activity by enhancing GABAergic inhibitory mechanisms. The decrease in IC-FA values observed in the current study may be interpreted as being related to the reorganization of axonal organization due to the effect of these inhibitory mechanisms [17].

The method developed for tinnitus treatment using VNS, based on the tonotopic model of tinnitus, also includes paired sound stimuli; it is stated that these sound stimuli initiate the reorganization of the auditory cortex to eliminate the source of the tinnitus perception [50]. However, a number of studies have also shown that tVNS alone can be successful in alleviating tinnitus symptoms without paired stimuli [36, 37]. The main rationale behind these initiatives is that tVNS modulates auditory and limbic areas, as demonstrated in neuroimaging studies conducted on normal subjects and tinnitus patients. Additional arguments include VNS’s positive effect on habituation, its antidepressant mechanism of action, and its effect on the autonomic nervous system [36]. tVNS also reduces autonomic nervous system imbalance associated with stress caused by tinnitus [38]. However, the fact that the effectiveness of tVNS in combination with matched auditory stimuli has not been evaluated in the current study can be considered a significant limitation. In addition, the relatively small number of participants also constitutes another limitation of the study. The most important limitation of this study is the absence of a control or sham-tVNS group, which prevents causal inference. Therefore, the improvements observed in THI and BDI scores and the decrease in inferior colliculus FA values cannot be conclusively attributed to tVNS treatment. These changes should be interpreted as exploratory and potentially suggestive of neuromodulatory effects rather than evidence of clinical efficacy. Future studies incorporating sham-controlled and randomized designs are required to determine whether the observed findings reflect true treatment-related changes or non-specific factors such as placebo effects, natural symptom variability, or repeated testing.

## Conclusion

The findings of this study suggest that a ten-session tVNS treatment may have the potential to reduce the burden of tinnitus on daily life and depression levels. As the first study to evaluate tVNS effects using DTI, these findings contribute novel insights into the neural mechanisms underlying tinnitus modulation. The results demonstrate that DTI can be used to objectively evaluate the effect of tinnitus treatment, complementing subjective assessment methods. Further investigation of tVNS for potential neuromodulatory changes is recommended. Future research involving larger, randomized controlled trials and extended follow-up periods is essential to validate these results and further elucidate the long-term impact of tVNS on auditory and emotional processing in patients with tinnitus.

## Data Availability

Upon request, the authors are willing to share the data associated with this research.
